# High-Density Microfluidic Chip with Vertical Structure for Digital PCR

**DOI:** 10.3390/s25175379

**Published:** 2025-09-01

**Authors:** Peng Sun, Huaqing Si, Gangwei Xu, Dongping Wu

**Affiliations:** 1 School of Information Technology, Luoyang Normal University, Luoyang 471934, China; sunpeng1@lynu.edu.cn; 2 State Key Laboratory of ASIC and System, School of Microelectronics, Fudan University, Shanghai 200433, China; 17112020035@fudan.edu.cn; 3 Shanghai Turtle Technology Company Limited, Shanghai 200439, China; 18112020050@fudan.edu.cn

**Keywords:** digital PCR, vertical structure, microfluidic chip, dynamic range, detection accuracy

## Abstract

Digital PCR, as a nucleic acid absolute quantification method at the single-molecule level, has been widely applied in early cancer screening, single-cell analysis, and other biomedical fields. However, existing digital PCR methods still suffer from high costs, complex operations, and low detection dynamic range, which limit their applications. In the study, we developed a microfluidic chip-based digital PCR with a high-density vertical structure using PDMS (polydimethylsiloxane) flexible material. The chip features a three-layer structure of glass–PDMS–glass, with the PDMS structural layer containing 30,000 reaction chambers, each with a volume of 0.713 nL. This vertical-structured chip can increase the total volume and the total number of chambers by 50% without changing the chip area and chamber volume, thereby significantly enhancing dynamic range and sensitivity of the chip detection. This chip is theoretically capable of achieving a nucleic acid detection dynamic range close to 10^5^. Moreover, the digital PCR quantitative detection results of five different concentrations of serially diluted KRAS plasmid DNA templates using this chip also validated the accuracy and reliability of the nucleic acid quantitative detection results. The vertical-structured digital PCR chip, with its simple manufacturing process, uniform and stable sample partitioning, wide detection dynamic range, and low cost, will promote the widespread application of digital PCR.

## 1. Introduction

Digital PCR (dPCR), as a nucleic acid absolute quantification method at the single-molecule level, has higher detection accuracy and sensitivity than quantitative real-time PCR (qPCR), attracting widespread attention in the field of biomedical detection [1,2,3,4]. Digital PCR works by partitioning the sample to be tested into thousands or even more independent reaction units, each of which contains one, several, or no target molecules [5]. Subsequently, independent PCR amplification reactions are carried out in each reaction unit. By counting the number of positive units with fluorescence signals after amplification and calculating the initial concentration of the sample according to the Poisson distribution formula, precise quantification of the sample is achieved [6,7]. For digital PCR technology, quantitative nucleic acid detection can be achieved by counting the number of positive units with fluorescence signals, without relying on standard curves, and it has higher accuracy and sensitivity. Like droplet microfluidic technology [8,9,10,11], digital PCR has broad application prospects in biomedical fields such as AMR diagnostics, single-cell analysis, early cancer screening, and clinical treatment [12,13,14,15,16,17].

Based on the differences in sample partitioning methods, existing digital PCR technologies are mainly divided into droplet-based digital PCR (ddPCR) [18,19,20,21,22,23] and chip-based digital PCR (cdPCR) [24,25,26]. Droplet-based digital PCR can quickly generate many droplet units and has the advantage of a high dynamic range [19]. However, the process of droplet generation, transfer, amplification, and detection involves multiple steps, resulting in higher equipment costs [27]. Chip-based digital PCR, also known as micro-chamber digital PCR, partitions the liquid using physical chambers and offers higher stability. Moreover, chip-based digital PCR typically allows for the intuitive acquisition of amplification results using a fluorescence microscope, which is more convenient and rapid [28,29].

In recent years, with the development of micro- and nanofabrication technologies and microfluidics, chip-based digital PCR has seen rapid growth [30,31,32]. The BioMark platform, developed by Fluidigm, is the earliest chip-based digital PCR system based on micro-channels and micro-valves [33]. However, this system requires complex micro-valves and external pump devices to achieve sample partitioning, which severely limits its application scope. To eliminate the need for external valves and control pumps, various chip-based microfluidic digital PCR platforms based on different design principles have been successively introduced, including micro-trap chips [34], sliding chips [35], self-digitizing chips [36], and rotary disk digital PCR platforms [37]. Each design has its own advantages, but all of the above digital PCR platforms require complex microfabrication or operational procedures to achieve sample partitioning. Subsequently, Zhu et al. [38] proposed a microfluidic dPCR chip based on PDMS, utilizing the negative pressure generated by the degassing of the flexible PDMS material as the driving force for sample loading, thereby simplifying the operational procedure. Similarly, Fu et al. [39] introduced a simple and reliable micro-chamber dPCR chip based on PDMS material. These dPCR studies have reduced the complexity of operation. However, digital PCR typically requires a good many small-volume reaction units to achieve a high detection dynamic range [24]. In common laboratories or resource-limited environments, reducing costs while enhancing the applicability of digital PCR detection is an important challenge [40].

To address the limitations of existing digital PCR platforms, we propose a high-density chip-based digital PCR using PDMS flexible material. This digital PCR chip features a vertical structural design, with the micro-chambers arranged above the micro-channels. Compared with the arrangement in which micro-chambers are positioned on either side of the micro-channels, the vertical structure allows for more-compact chamber arrangement and higher chamber density. This enhances the dynamic range of detection without increasing the chip area. The chip uses PDMS and glass as the main structural materials, offering advantages such as simple fabrication, low cost, and high density. The digital PCR quantification results for five different concentrations of KRAS plasmid DNA templates, which were serially diluted, also validated the accuracy and reliability of nucleic acid quantification using this chip. The high-density microfluidic chip with a vertical structure is conducive to further promoting the application and development of digital PCR technology in a broader range of biomedical fields.

## 2. Materials and Methods

### 2.1. Chip Design and Fabrication

**Figure 1:**
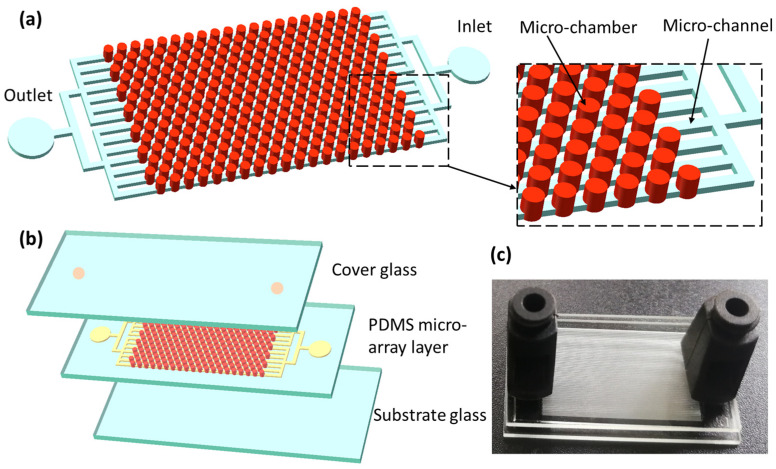
Schematic of the high-density microfluidic chip with vertical structure. (**a**) The dPCR chip structural design. The PDMS structure layer of the chip consists of channels and chambers. The size of the chamber is 120 μm in height, 87 μm in diameter. The height of the channel is 30 μm, 50 μm in width. (**b**) The layered structure of the chip comprising a PDMS micro-array layer, a glass substrate layer, and a glass coverslip layer. (**c**) Physical image of high-density vertical microfluidic dPCR chip.

Figure 1a,b show the schematic diagrams of the high-density dPCR chip structure proposed in this study. The chip is made up of a three-layer structure of glass–PDMS–glass, composed of a glass substrate, a PDMS structural layer, and a glass cover plate from bottom to top. The PDMS structural layer of the digital PCR chip includes micro-channels and cylindrical micro-chambers. The micro-chambers are arranged above the micro-channels, thereby eliminating the area occupied by the micro-channels on the chip and achieving higher chamber density. The chip contains a total of 30,000 cylindrical micro-chambers, each with a diameter of 87 µm and a height of 120 µm, forming a single micro-chamber volume of 0.713 nL. The fabricated digital PCR chip is shown in Figure 1c, with overall dimensions of 25 mm × 15 mm.

**Figure 2:**
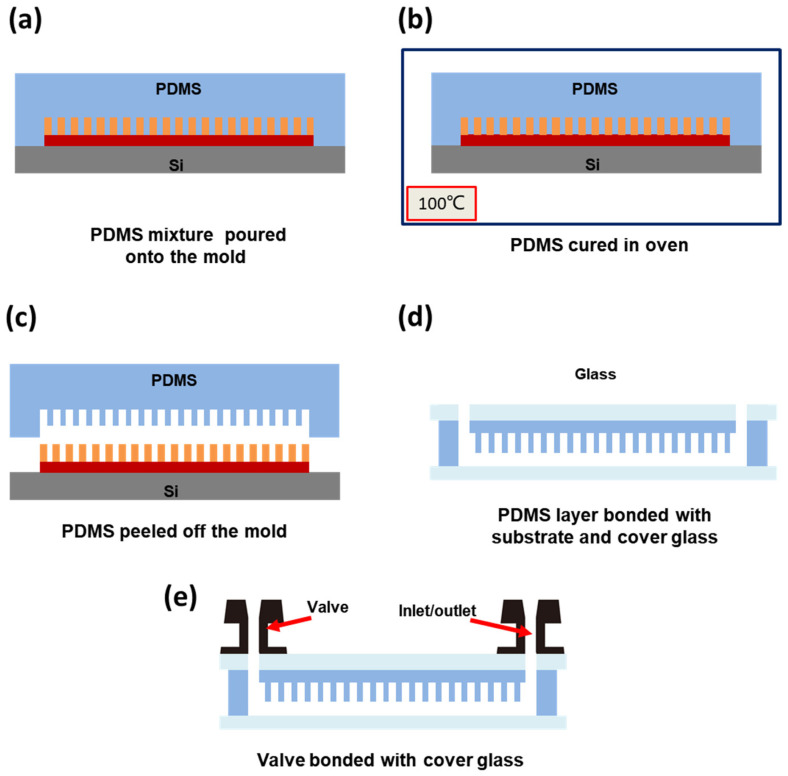
The preparation process of the chip. (**a**) Pour the PDMS mixture onto the silicon wafer mold. (**b**) Place the PDMS structural layer in an oven and heat it to cure. (**c**) Peel off the PDMS structural layer from the mold. (**d**) PDMS structural layer bonded with substrate and cover glass. (**e**) Valve bonded with cover glass.

The preparation process of the high-density microfluidic chip with a vertical structure mainly includes two parts: the fabrication of the silicon-based mold for the PDMS structural layer and the fabrication of the chip. The silicon-based mold for the PDMS structural layer was prepared using a multi-layer lithography process. Then, the chip was fabricated by bonding the PDMS structural layer to the glass substrate. To fabricate the silicon-based mold for the PDMS structural layer, an SU-8 negative photoresist (MicroChem, Westborough, MA, USA) was coated on a 4-inch silicon wafer, and a two-layer lithography process was employed to obtain the mold. The detailed fabrication process of the silicon mold is shown in Figure S1. After the mold was prepared, the chip fabrication process began; the production process of the chip is shown in Figure 2. Firstly, as shown in Figure 2a, PDMS monomer (Dow Corning, Midland, MI, USA) was mixed with the curing agent at a mass ratio of 10:1. Then, Triton X-100 (Sigma Aldrich, St. Louis, MO, USA) surfactant, at a mass ratio of 0.5%, was added to the mixture. It can make the surface of the mixture more hydrophilic and facilitate entry of the reagent into the chamber. Subsequently, the mixture was degassed in a vacuum chamber and then poured onto the mold and left undisturbed for 30 min. Next, as shown in Figure 2b, the silicon wafer with the PDMS mixture was placed horizontally into an oven at 100 °C and heated for 30 min to cure the PDMS. After curing, as shown in Figure 2c, the PDMS structural layer was carefully peeled off from the mold. Holes with a diameter of approximately 1.5 mm were then punched at the inlet and outlet ports using a punch tool. As shown in Figure 2d, the PDMS structural layer and the glass substrate were treated with plasma to facilitate bonding. Once bonded together, the chip was placed in an oven at 80 °C for 30 min to strengthen the bonding interface. Finally, as shown in Figure 2e, two rubber valves with channels were bonded to the inlet and outlet ports on the glass cover.

### 2.2. Microfluidic Chip Operation

**Figure 3:**
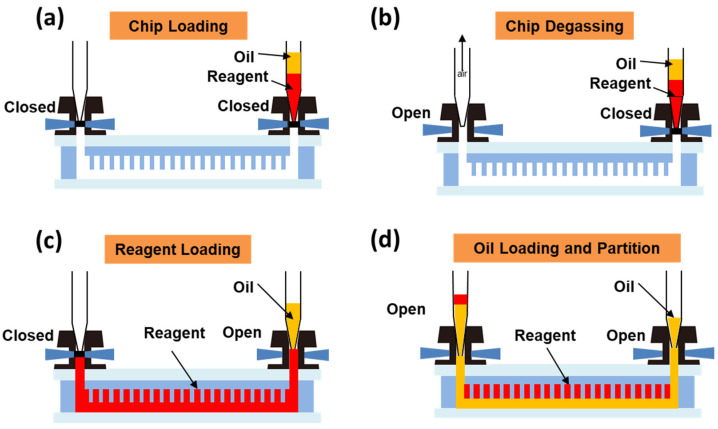
Sample loading process of the dPCR chip. (**a**) Mount the chip onto the automated sample loading device. (**b**) Perform degassing treatment on the chip. (**c**) Air pressure difference pushes reagent into the chip chambers and channels. (**d**) Oil phase enters the chip channels, reagent in each micro-chamber is separated by the oil phase in the channels.

We used a digital PCR automated sample loading device (Turtle Tech Ltd., Shanghai, China) to complete the sample loading operation. The name of the oil used in the experiment is dPCR Sealing Oil (Turtle Tech Ltd., Shanghai, China); its main component is silicone oil. First, the chip was placed on the chip holder of the automated sample loading device. Then, using a pipette, 30 µL of oil phase and 25 µL of reagent were sequentially drawn into the pipette tip, as shown in Figure 3a; the pipette tip containing the oil phase and PCR reaction reagent was inserted into the sample inlet, and an empty pipette tip was inserted into the sample outlet to collect the excess oil phase and reagent that would be expelled. Subsequently, the micro-valves at the sample inlet and outlet were closed. The second step was chip degassing, shown in Figure 3b; the micro-valve at the sample outlet was opened while keeping the valve at the sample inlet closed, and a vacuum pump was used to evacuate the chip to a pressure below 0.5 kPa, a process that takes approximately 20 s to complete. The third step was reagent loading, as shown in Figure 3c; the sample outlet was closed first, and then the sample inlet was opened. The pressure difference between the inside and outside of the chip drove the reagent to rapidly enter the micro-channels and micro-chambers within the chip. After about 30 s, the liquid phase filled the micro-chambers and micro-channels of the chip. The final step was oil-phase separation, as shown in Figure 3d; after the reaction reagent filled the chip, the micro-valve at the outlet was opened, and a positive pressure (131 kPa) was quickly applied at the sample inlet. This pushed the oil phase into the micro-channels of the chip, forcing the reagent in the micro-channels to the sample outlet pipette tip. At this point, the reaction reagent in each micro-chamber was separated by the oil phase in the channels, forming independent digital PCR reaction units.

### 2.3. PCR Conditions

The components of the reaction reagents used in the digital PCR amplification reaction include PCR pre-mix, primers, templates, and probes, which should be pre-mixed before loading into the dPCR system. To verify the nucleic acid detection performance of the designed digital PCR chip, a series of KRAS wild-type DNA templates with concentrations differing by a factor of 10 were prepared, with dilution concentrations ranging from 6.5 × 10^0^ to 6.5 × 10^4^ copies/μL. The dPCR reaction mixture in this experiment included 10× BioDigital dPCR Mix (3 μL), KRAS forward primer (1 μL) (400 nM), KRAS reverse primer (1 µL) (400 nM), KRAS probe (1 μL) (400 nM), serially diluted template (3 µL), and nuclease-free water (21 µL). The digital PCR amplification reaction was performed on a flat digital PCR cycler (Turtle Tech Ltd., Shanghai, China). The thermal cycling program for the PCR amplification reaction was set as follows: 50 °C for 10 min to solidify the oil phase and 95 °C for 10 min to activate the Taq DNA polymerase; then 45 thermal cycles (95 °C for 20 s, 58 °C for 40 s) were performed to amplify the target DNA. The entire PCR reaction time was about 80 min.

### 2.4. Data Acquisition and Analysis

The bright-field images of the digital PCR chip were captured using an optical microscope (XDS-800C, Caikon, Shanghai, China). The fluorescence images of the chip were captured and analyzed by BioDigital dPCR reader (Turtle Tech Ltd., Shanghai, China). The fluorescence was excited at 620 nm, and the light was detected by a CCD image sensor through a long-pass filter at 660 nm.

## 3. Results and Discussion

### 3.1. Effect of High-Density Vertical-Structure Design on Sensitivity and Dynamic Range of dPCR Chip

The sensitivity and dynamic range of digital PCR are vital performance indicators. The dynamic range of detection refers to the range of sample concentrations that the PCR system can quantify. The sensitivity of digital PCR detection refers to the lowest target template concentration that can be detected, which is also the lower limit of detection. The dynamic range of detection for a digital PCR platform is determined by the upper and lower limits of detection. The upper limit of detection is mainly up to the volume of a single chamber and the total number of chambers, while the lower limit of detection is up to the total reaction volume. Therefore, increasing the total reaction volume and the number of chambers is a useful way to improve detection sensitivity and broaden the dynamic range of detection. However, for digital PCR platforms, having a larger total reaction volume and many more chambers usually requires a larger chip area. Under the premise of not increasing the chip area, increasing the chamber density to boost the number of chambers and the total reaction volume is a very effective solution.

The high-density microfluidic chip with a vertical structure proposed in this study has its micro-chambers arranged above the micro-channels, eliminating the area occupation of micro-channels on the chip. This structure can significantly increase the chamber density on the chip, thereby increasing the number of chambers and the total reaction volume and improving the spatial utilization rate of the chip. Table 1 shows the comparison results of vertical-structure chips and horizontal-structure chips with the same chip area and the same micro-chamber size. As can be seen from the table, compared with the horizontal-structure chip, under the condition of the same chip area and micro-chamber size, the number of chambers on the chip can be increased from 20,000 to 30,000 by adopting the vertical-structure design. Compared with a horizontal-structure digital PCR chip, the vertical-structure digital PCR chip has a higher total chamber volume and a greater number of chambers. Therefore, the dynamic range of the vertical-structure digital PCR chip reaches more than 1.5 times that of the horizontal-structure chip. In summary, the vertical-structure digital PCR chip we designed significantly improves the detection sensitivity and increases the dynamic range under the same chip area and chamber volume conditions.

### 3.2. Sample Partition of dPCR Chip

**Figure 4:**
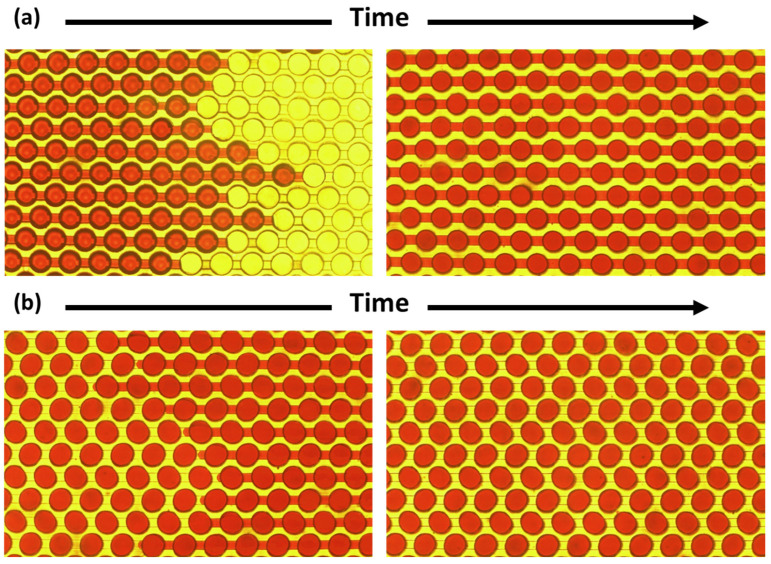
Optical micrograph of the process of loading red dye solution and oil into the dPCR chip. (**a**) The red dye solution was driven into the micro-channels and micro-chambers. (**b**) The red dye solution was totally separated into each chamber by the oil phase flowing into the chip.

The digital PCR chip employs a negative-pressure self-suction method for loading reagents, taking advantage of the higher gas solubility and better gas permeability of PDMS material. The reagent loading operation of the chip requires the use of an automatic dPCR chip loader. To facilitate observation, a red dye solution is used instead of a colorless reagent to demonstrate this process and verify the liquid-separation effect. First, the digital PCR chip was placed in the chip slot of the loading device. Then, using a pipette, 30 μL of oil phase and 25 μL of red dye solution were successively drawn into the pipette tip, which was then inserted into the inlet. After degassing the chip, the inlet valve was opened. As shown in Figure 4a, the red dye solution was rapidly driven by the negative pressure into the micro-channels and micro-chambers; after about ten to fifteen seconds, the red dye solution was completely absorbed into the chip, filling the micro-chambers and micro-channels. As shown in Figure 4b, the oil phase was then pushed into the micro-channels, displacing the red dye solution in the channels; as the red dye solution in the channels was completely displaced by the oil phase and expelled into the pipette tip at the outlet, the red dye solution in the micro-chambers above the channels was completely separated by the oil phase in the channels below, forming independent reaction units. It can be seen that the designed digital PCR chip can complete the reagent loading process in a simple and rapid manner.

### 3.3. Uniformity Analysis of the Chip

**Figure 5:**
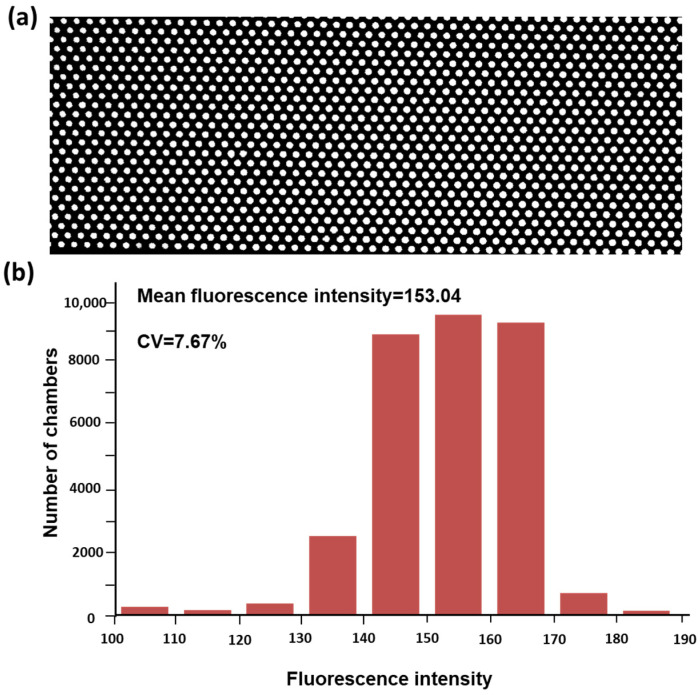
Diagram of the reagent loading uniformity analysis of the high-density microfluidic chip with vertical structure. (**a**) Fluorescent image of micro-chambers filled with fluorescence reagent. (**b**) Statistical analysis of fluorescence intensity distribution of all micro-chambers.

To verify the uniformity of reagent loading in the designed digital PCR chip, fluorescent dye was used to load the chip, and then the fluorescence intensity distribution in each chamber was tested to characterize the uniformity of the loading process. First, we loaded the chip with the fluorescent dye as a substitute for the reagent. Then, we obtained fluorescence images of the chip using the BioDigital dPCR (BioAnalysiserQing) reader and measured the fluorescence intensity values of each chamber using ImageJ (Windows 64-bit Java 8). Figure 5a shows the fluorescence micrograph of the chip after the fluorescent dye was dispersed. Figure 5b illustrates the distribution of fluorescence intensity in the micro-chambers, with the *x*-axis representing the fluorescence intensity values and the *y*-axis representing the number of micro-chambers corresponding to different fluorescence intensity values. The statistical analysis of the fluorescence intensity distribution in the micro-chambers indicates that the majority of the chambers have fluorescence intensity values between 140 and 170. The average fluorescence intensity was calculated to be 153.04, with a relative standard deviation of 7.67%. It shows that the reagent is evenly allocated within the chambers, ensuring high consistency in the loading process.

### 3.4. DNA Quantification Analysis by dPCR

**Figure 6:**
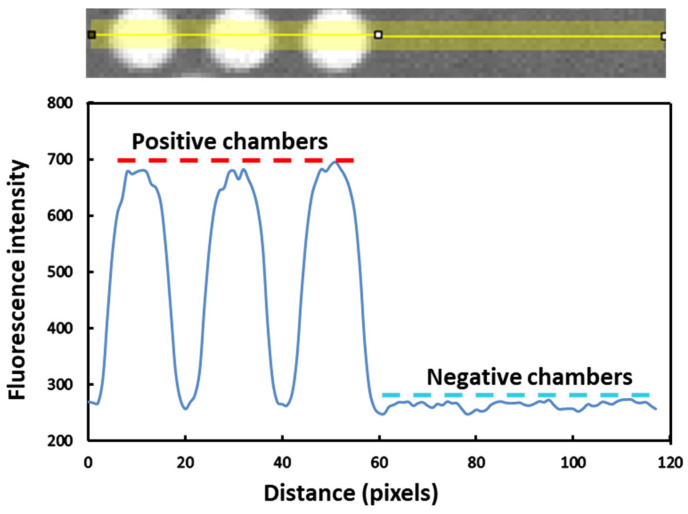
Fluorescence intensity comparison between positive and negative chambers after PCR amplification reaction. The yellow line represents the statistical area of fluorescence intensity. The blue curve indicates the change in fluorescence intensity corresponding to the chip position above.

The most vital application of dPCR is the absolute quantification of nucleic acid molecules without relying on a standard curve. In this study, we used wild-type KRAS DNA templates for experimental validation. Mutations in codons 12 and 13 of the KRAS gene are highly prevalent in colorectal cancer and pancreatic cancer and especially in the early stages of colorectal cancer, pancreatic cancer, and lung cancer. Therefore, the detection of KRAS mutations is very important for the early diagnosis of cancer [41,42,43]. Before conducting the quantitative experiments, we first verified the ability of the digital PCR chip to distinguish positive chambers. A wild-type KRAS DNA template with a concentration of 6.5 × 10^3^ copies/μL was used as the validation sample. After loading the digital PCR chip and performing amplification, we selected a CY5 fluorescent probe to compare the differences in fluorescence intensity between positive and negative chambers after PCR amplification. Figure 6 shows the comparison results of fluorescence intensity between positive and negative chambers. The blue curve in the figure represents the fluorescence intensity value corresponding to different horizontal positions of the yellow lines on the chip. It can be seen that the fluorescence intensity of the positive chambers is more than twice that of the negative chambers. Therefore, the chip can easily distinguish between positive and negative chambers, meeting the detection requirements.

To verify the nucleic acid quantification ability of the vertical-structure digital PCR chip within its dynamic range, we conducted quantitative experiments using KRAS wild-type DNA templates with known concentrations. In the experiment, five different concentrations of serially diluted KRAS wild-type DNA templates were prepared for digital PCR reactions, with concentrations ranging from 6.5 × 10^0^ to 6.5 × 10^4^ copies/μL. The five concentration gradients of samples were loaded and amplified separately. The fluorescence signals at the endpoint of the reaction were obtained using the BioDigital dPCR reader and subjected to statistical analysis. Each concentration of the sample was tested in triplicate. For digital PCR, the Poisson distribution principle can be utilized to calculate the concentration of the target DNA template in the PCR mixture based on the number of positive chambers in the endpoint fluorescence image. The calculation formula for concentration C is as follows:

(1) $$C=-\frac{\ln(1-\frac{d}{n})}{V_{d}}$$

Here, n represents the total number of micro-chambers in the dPCR chip that participate in the reaction; d is the number of positive chambers; $\frac{d}{n}$ is the proportion of positive chambers; and $V_{d}$ is the volume of a single chamber (μL). The concentration of the target DNA template for the five different concentrations of samples was calculated using the above formula. The statistical data of the digital PCR assay are shown in the Supplementary Materials (Table S1).

**Figure 7:**
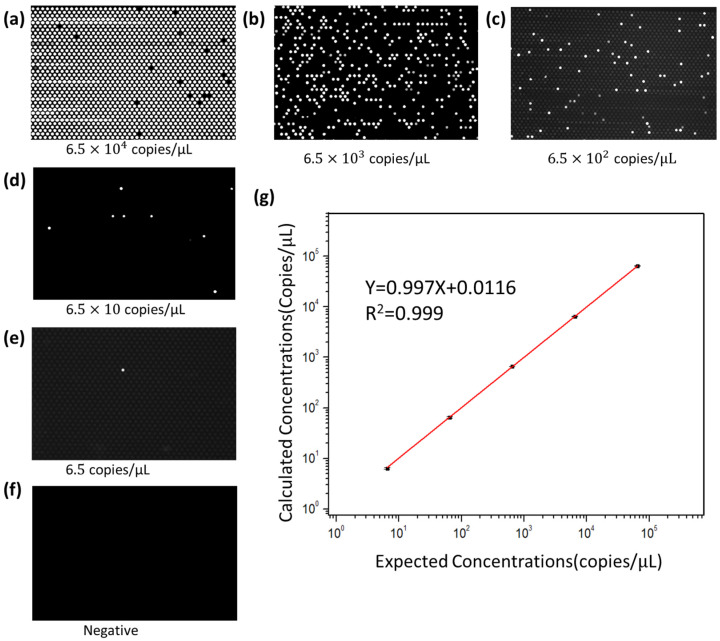
Digital PCR on the high-density microfluidic chip with vertical structure with different concentration of KRAS plasmid DNA template. (**a**–**e**) Digital PCR on the chip with a serial dilution of the target DNA template ranging five orders of magnitude from 6.5 × 10^4^ to 6.5 × 10^0^ copies/μL. (**f**) The negative control with no target template loaded. (**g**) The linear relationship between the expected DNA concentration and the calculated concentration.

Figure 7a–e show the fluorescence imaging photos of the chip after PCR amplification for samples with different concentrations. It can be shown from the figures that, with the concentration of the DNA template decreasing after dilution, the proportion of positive chambers also correspondingly decreases. Figure 7f shows the results of the negative control, in which no positive chamber is present after amplification, indicating that there is no contamination during the reaction process. Figure 7g presents the “theoretical concentration—experimentally calculated concentration” curve drawn based on the experimental results. The linear fitting curve shows that the DNA concentration measured using the vertical-structure digital PCR chip can match the theoretical DNA concentration very well (R^2^ = 0.999). The experiment demonstrates that the high-density microfluidic chip with a vertical structure designed in this study provides accurate and reliable results for nucleic acid quantification.

## 4. Conclusions

This study proposes and fabricates a high-density microfluidic chip with a vertical structure for digital PCR. Compared with the horizontally arranged microfluidic digital PCR, the vertical-structure chip can increase the total volume of reaction chambers and the total number of chambers by 50% without changing the chip area and the size of the chamber volume. This greatly enhances the detection sensitivity and dynamic range of the chip. The designed chip theoretically achieves a nucleic acid detection dynamic range close to 10^5^. Moreover, the chip is based on a three-layer structure of glass–PDMS–glass and is fabricated using PDMS soft lithography and plasma bonding techniques. The fabrication process is simple and cost effective. The chip employs a negative-pressure-driven loading method, which is easy to operate and reliable. This study experimentally verified the loading effect of the chip, and the results showed that the chip can effectively partition the samples. The uniformity of the fluorescence signal after chip amplification was tested, and the results indicated that the reagent is evenly distributed in each chamber of the chip, with uniform loading that meets the detection requirements of digital PCR. The digital PCR quantitative detection results of five different concentrations of serially diluted KRAS plasmid DNA templates also verified the accuracy and reliability of the nucleic acid quantification by the chip. In summary, the high-density vertical-structure digital PCR chip designed and fabricated in this study is characterized by its simple fabrication process, uniform and stable sample partitioning, wide detection dynamic range, and low cost. These features contribute to the expansion of the application fields of digital PCR. We will strive to expand the application of the digital PCR chip in fields such as AMR diagnostics, single-cell analysis, and early cancer screening in the future.

## Figures and Tables

**Table 1:** Comparison of vertical-structured dPCR chip and planar-structured dPCR chip in detection sensitivity and dynamic range.

Chip Structure	Number of Chambers	Total Volume (μL)	Lower Limit of Detection (Copies/μL)	Upper Limit of Detection (Copies/μL)	Dynamic Range
Vertical structure	30,000	21.39	1.4	1.29 × 10^5^	9.21 × 10^4^
Planar structure	20,000	14.26	2.1	1.24 × 10^5^	5.9 × 10^4^

## Data Availability

All datasets presented in this study are included in the article.

## Supplementary Materials

The following supporting information can be downloaded at https://www.mdpi.com/article/10.3390/s25175379/s1: Figure S1: Schematic illustration of the fabrication process of the silicon mold; Table S1: The vertical-structure digital PCR statistical analysis result of the KRAS plasmid template.
