# Supplementary material for: High-Density Microfluidic Chip with Vertical Structure for Digital PCR
Source: Sensors (Basel). 2025 Sep 1;25(17):5379. doi: 10.3390/s25175379 (PMC12431553; doi:10.3390/s25175379)
Supplement: Supplementary file 1 [file sensors-25-05379-s001.zip › sensors-3824775-supplementary.pdf]

Supporting Information
High-Density Microfluidic Chip with Vertical Structure for Digital PCR

Peng Sun [1,†], Huaqing Si [2,†], Gangwei Xu [3] and Dongping Wu [2,*]

[1] School of Information Technology, Luoyang Normal University, Luoyang 471934, China

[2] State Key Laboratory of ASIC and System, School of Microelectronics, Fudan University, Shanghai 200433, China

[3] Shanghai Turtle Technology Company Limited, Shanghai 200439, China

[*] Correspondence: dongpingwu@fudan.edu.cn

[†] These authors contributed equally to this work.

The preparation process of the silicon mold was shown in Figure S1. Photolithography masks were prepared for the channel and chamber layer of the chip before fabrication. The cleaned silicon wafer was placed on a hot plate at 180°C and baked for 30 minutes. Afterwards, 30 μm thick SU8 was coated on the surface of the silicon wafer. The spin coating process was to spin the silicon wafer at 500 rpm for 10 seconds, followed by 40 seconds at 4300 rpm. The silicon wafer was then placed on the UV lithography machine (NXQ4006 Mask Aligner, California, USA) for exposure treatment, and after development, the channel layer structure was completed. Then, 100 μm thick SU8 was coated onto the silicon wafer. The spin coating process was to spin the silicon wafer at 500 rpm for 10 seconds, followed by 40 seconds at 1400 rpm. The alignment mark on the mask was aligned with the alignment mark on the first layer channel structure on the silicon wafer, which was exposed and developed to complete the fabrication of the chamber layer structure. After that, the mold was baked at 170 °C for 30 minutes, and then the mold was finished.

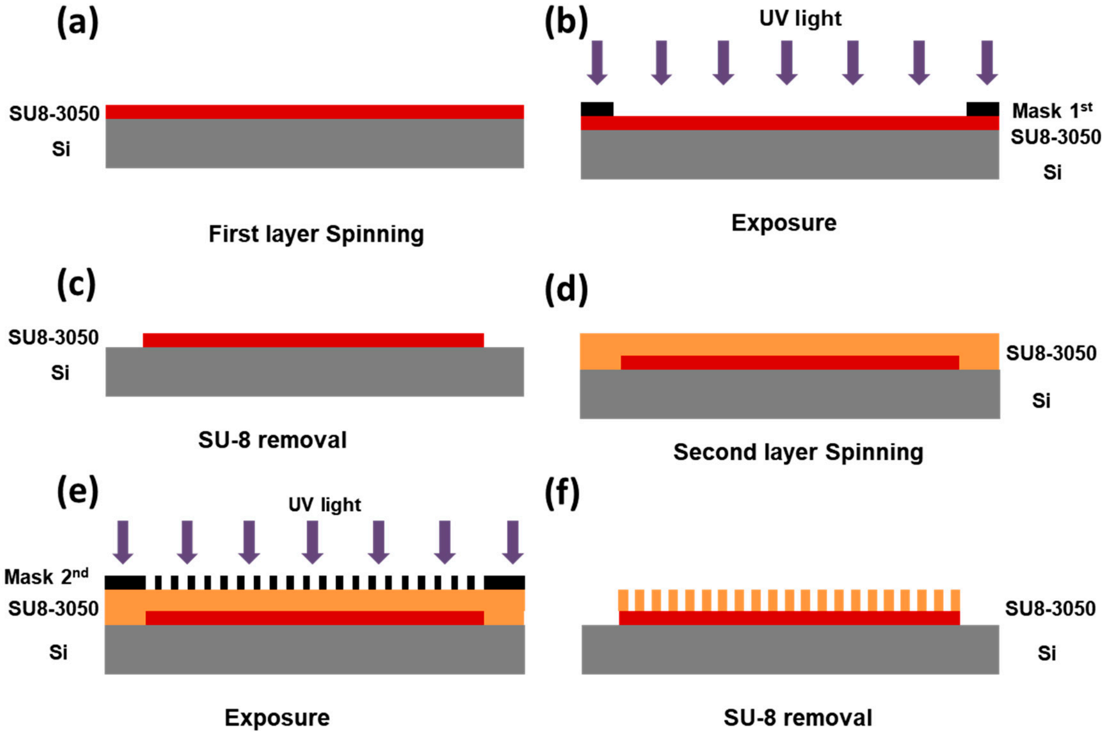

Figure S1. Schematic illustration of the fabrication process of the silicon mold. (a) SU8 was coated on the silicon wafer. (b) The wafer was exposed using a mask aligner. (c) Developing to form the microchannel. (d) SU8 was coated again. (e) The wafer was exposed after aligned with the first layer structure. (f) Developing to form the micro-chambers and finishing the mold.

Table S1. The vertical structure digital PCR statistical analysis result of the KRAS plasmid template

Expected concentration (copies/μL)	Experiment 1 C_1(copies/μL)	Experiment 2 C_2(copies/μL)	Experiment 3 C_3(copies/μL)	Average (calculated concentration) (copies/μL)	STDEV
6.5×10^0	6.55×10^0	6.08×10^0	6.55×10^0	6.39×10^0	0.27
6.5×10^1	6.23×10^1	6.51×10^1	6.70×10^1	6.48×10^1	2.36
6.5×10^2	6.53×10^2	6.55×10^2	6.61×10^2	6.56×10^2	4.16
6.5×10^3	6.38×10^3	6.59×10^3	6.54×10^3	6.50×10^3	109.69
6.5×10^4	6.51×10^4	6.43×10^4	6.35×10^4	6.43×10^4	800

* Where C_1, C_2 and C_3 are the calculated concentrations of the three repeated experimets, respectively.
